# Prognostic value of the creatinine-to-albumin ratio for 28-day mortality in patients with sepsis and diabetes: integrating renal and nutritional status in the ICU

**DOI:** 10.3389/fnut.2026.1724997

**Published:** 2026-02-03

**Authors:** Jianzhu Zhou, Hui Qiu, Jiahui Li, Jiamiao Liu, Chengxian Guo

**Affiliations:** Center of Clinical Pharmacology, The Third Xiangya Hospital, Central South University, Changsha, China

**Keywords:** creatinine-to-albumin ratio, diabetes mellitus, ICU mortality, renal and nutritional status, sepsis

## Abstract

**Background:**

Patients with sepsis and diabetes exhibit complex pathophysiology that greatly increases the risk of adverse outcomes. This study aimed to investigate the association between CAR and 28-day all-cause mortality among intensive care unit (ICU) patients with sepsis and diabetes mellitus.

**Methods:**

In this retrospective cohort study based on the eICU-CRD, we analyzed 1,800 adult patients with sepsis and diabetes mellitus, who were stratified into quartiles by admission CAR. Survival was assessed by Kaplan–Meier analysis. Covariates were selected via LASSO and stepwise regression. Multivariate Cox models evaluated CAR’s independent association with mortality. Restricted cubic splines explored nonlinear relationships. ROC analysis and subgroup analyses were performed.

**Results:**

Multivariate Cox proportional hazards analysis indicated that CAR was significantly correlated with the 28-day mortality risk in the ICU. Patients in the highest quartile (Q4) had a 4.68-fold greater risk of death compared with those in the lowest quartile [HR 4.68, 95% CI 2.55–8.61, *p* < 0.001]. CAR had an AUC of 0.664 for mortality prediction and was associated with a higher mortality risk in the norepinephrine group [HR 8.26, 95% CI 2.55–26.76, *p* < 0.001]. Higher CAR was also associated with a longer ICU length of stay (*β* = 1.06, *p* = 0.006).

**Conclusion:**

CAR was associated with increased 28-day mortality in ICU patients with sepsis and diabetes. These findings suggest that CAR may be useful for prognosis assessment and risk stratification.

## 1 Background

Sepsis is a life-threatening organ dysfunction resulting from a dysregulated host response to infection (1). As a heterogeneous syndrome, variations in infection sources and host comorbidities contribute to diverse prognostic outcomes (2). Patients with diabetes exhibit a markedly increased susceptibility to infection, and sepsis complicated by diabetes involves more complex pathophysiological mechanisms (3). Diabetes notably elevates the risk of multi-organ failure in sepsis through chronic inflammatory states, endothelial dysfunction, and impaired immune regulation (4, 5). Previous studies have demonstrated that the coexistence of sepsis and diabetes is associated with a significantly higher 28-day all-cause mortality compared with sepsis without diabetes (6–9). Early and accurate assessment of sepsis severity in patients with diabetes may facilitate improved clinical decision-making and reduce mortality. However, established scoring systems such as the Sequential Organ Failure Assessment (SOFA) (10) and Systemic Inflammatory Response Syndrome (SIRS) (11) require extensive data collection prior to evaluation, and not all parameters are readily available, potentially delaying severity assessment and missing the optimal therapeutic window (12, 13). Consequently, there is an urgent need to identify simpler, more rapid, and accessible biomarkers to enhance risk stratification in sepsis patients with diabetes.

Previous studies have demonstrated that serum creatinine (Cr) and albumin (Alb) levels are independently associated with the severity and prognosis of sepsis and diabetes, respectively (14–17). Acute kidney injury represents a critical organ dysfunction in sepsis (18), while patients with diabetes frequently exhibit renal insufficiency secondary to microvascular damage (19). Serum creatinine serves as a reliable indicator of renal function and constitutes an important prognostic biomarker in both conditions (20). Metabolic and inflammatory dysregulation are hallmark features shared by patients with sepsis and diabetes (5). Albumin functions as a prognostic marker reflecting nutritional status, inflammatory burden, and hepatic synthetic capacity (21). Notably, prior research has identified the serum creatinine-to-albumin ratio (CAR) as a predictor of adverse outcomes in critically ill patients (22–24). Nonetheless, the prognostic utility of CAR within specific sepsis-related comorbidity subgroups remains insufficiently investigated, particularly among patients with diabetes characterized by metabolic disturbances and distinct immune profiles (25). CAR amplifies the signal of changes in a single indicator, integrating two pathophysiological mechanisms (26), reflecting the extent of kidney injury and inflammation-nutritional status, and may possess higher predictive value in the prognosis of sepsis complicated by diabetes.

The association between the creatinine-to-albumin ratio (CAR) and prognosis in patients with sepsis and diabetes remains poorly understood. This study aims to investigate the relationship between CAR and 28-day all-cause mortality among patients with sepsis and diabetes admitted to the ICU. Insights gained from this research may facilitate early risk stratification in this high-risk population and contribute to the development of novel therapeutic strategies to improve clinical outcomes.

## 2 Methods

### 2.1 Data sources

The data utilized in this study were extracted from the eICU Collaborative Research Database (eICU-CRD), a large, multicenter intensive care unit database developed and maintained by the University of Pittsburgh and Philips Healthcare (27). This publicly accessible clinical database encompasses detailed information on more than 200,000 critically ill patients admitted to over 200 hospitals across the United States, including a diverse range of teaching and community hospitals, during the period from 2014 to 2015. As the dataset has been fully de-identified to ensure patient privacy, this study was exempt from informed consent and ethical approval requirements.

### 2.2 Inclusion and exclusion criteria

**Figure 1:**
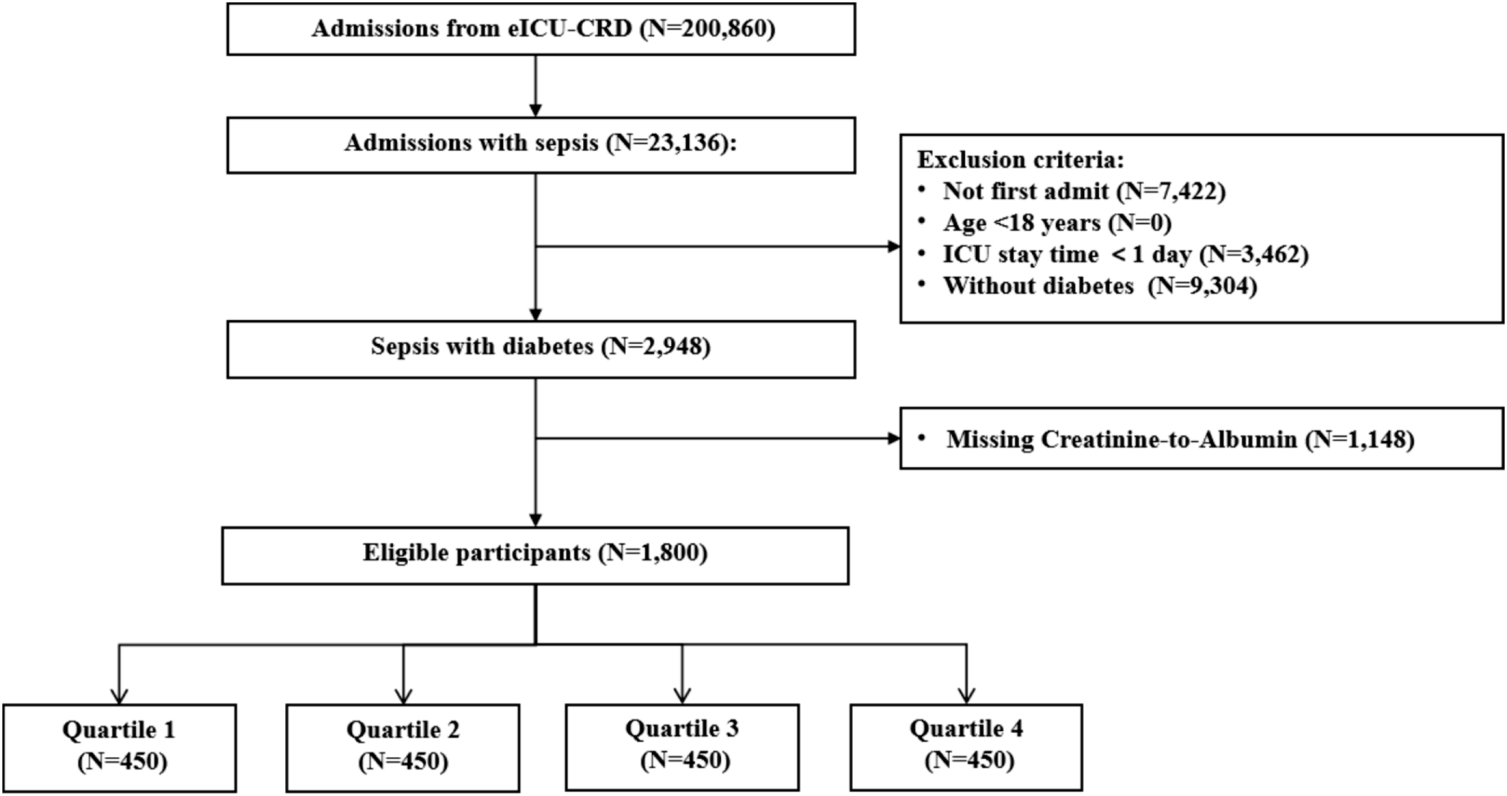
Patient screening and selection flowchart.

In this study, patients diagnosed with sepsis according to ICD-9 codes (995.91, 995.92, and 785.52) were included. Additionally, the “apacheAdmissionDx” field in the “admissionDx” table was queried to retrieve full path string of admission diagnosis for each patient’s unit stay, incorporating the original diagnostic information recorded by clinicians. The exclusion criteria were as follows: (1) patients who were not admitted to the ICU for the first time (only data from the first admission were extracted), (2) patients under 18 years of age, (3) patients with an ICU length of stay of less than 1 day, (4) patients lacking key baseline laboratory data (serum creatinine and serum albumin) on the first day of ICU admission, and (5) patients without a diagnosis of diabetes. Ultimately, 1,800 patients were included in this study and divided into four groups based on CAR quartiles (Figure 1).

### 2.3 Data collection

Data were extracted using Structured Query Language (SQL). Potential variables were divided into four major categories: (1) demographic data, including age, gender, body mass index (BMI), ethnicity, (2) comorbidities, including chronic kidney disease, hypertension, tumors, chronic obstructive pulmonary disease, and others; (3) laboratory indicators, including serum creatinine, albumin, bilirubin, blood glucose, and white blood cell count, and (4) Treatment measures, including norepinephrine, dialysis, and mechanical ventilation. To minimize potential bias, variables with more than 10% missing data were excluded from the analysis. For variables with less than 10% missing data, continuous variables were imputed using the median, while categorical variables were imputed using the mode.

### 2.4 Clinical outcomes

The primary endpoint of this study was 28-day all-cause mortality among patients admitted to the ICU, and the secondary endpoint was all-cause in-ICU mortality.

### 2.5 Statistical analysis

Continuous variables were described as medians with interquartile ranges (IQRs), while categorical variables were expressed as frequencies and percentages. For normally distributed continuous variables, comparisons between groups were performed using Student’s t-tests or one-way analysis of variance (ANOVA), whereas non-normally distributed variables were compared using the Mann–Whitney U test or Kruskal-Wallis test. Categorical variables were compared using the Pearson chi-square test or Fisher’s exact test, as appropriate. Kaplan–Meier curves were used to estimate survival probabilities across CAR quartiles, and differences were assessed using the log-rank test. Cox proportional hazards regression models were used to evaluate the association between CAR and the study endpoints, with results reported as hazard ratios (HRs) and 95% confidence intervals (CIs). Variable selection for multivariable adjustment was performed using least absolute shrinkage and selection operator (LASSO) regression to identify key variables, followed by stepwise regression to address multicollinearity. The final multivariable Cox model incorporated the selected covariates. CAR was modeled both as a continuous variable and categorically by quartiles, with the lowest quartile (Q1) as the reference. *p*-values for trend were calculated across quartiles. Restricted cubic splines (RCS) were used to assess potential nonlinear associations between CAR and ICU mortality. The predictive performance of CAR was compared with that of serum creatinine or albumin alone using the area under the receiver operating characteristic curve (AUC), with comparisons via DeLong’s test. Subgroup analyses were performed to explore heterogeneity across patient subgroups. Additionally, linear regression was employed to examine the relationship between CAR and ICU length of stay in survivors. All analyses were conducted using R software (version 4.4.0), and a two-sided *p*-value < 0.05 considered statistically significant.

## 3 Results

### 3.1 Baseline characteristics

A total of 1,800 patients with sepsis and diabetes were included in this study and stratified into four groups according to CAR quartiles. Baseline characteristics are presented in Table 1. The median age of the entire cohort was 60 years, with 942 (52.3%) male patients. The overall ICU mortality rate was 10.2%, and the median ICU length of stay was 2.78 days. Compared with patients in the lowest quartile (Q1), those in the higher quartiles (Q2-Q4) were generally older, had higher baseline BMI, white blood cell count, bilirubin, and blood urea nitrogen levels, received more norepinephrine and dialysis treatments, and had higher prevalence rates of chronic kidney disease and septic shock. As CAR increased across quartiles, both ICU length of stay (2.45 days vs. 2.59 days vs. 3.01 days vs. 3.40 days, *p* < 0.001) and ICU mortality rates (3.3% vs. 7.1% vs. 13.1% vs. 17.1%, *p* < 0.001) demonstrated a gradual increasing trend.

**Table 1:** Baseline characteristics of patients stratified by quartiles of the serum CAR.

Characteristics	Overall (*N* = 1,800)	Q1 (*N* = 450)	Q2 (*N* = 450)	Q3 (*N* = 450)	Q4 (*N* = 450)	*p*-value
Age, years, median (IQR)	68 (59–77)	65.00 (56.00–73.00)	70.00 (61.00–80.00)	70.50 (61.00–78.00)	66.00 (58.00–74.00)	<0.001
Male, *n* (%)	942 (52.3%)	198 (44.0%)	228 (50.7%)	256 (56.9%)	260 (57.8%)	<0.001
BMI, kg/m^2^, median (IQR)	30.03 (25.37–36.01)	29.23 (24.80–34.26)	29.97 (25.03–35.52)	30.84 (25.94–37.17)	30.45 (25.79–36.81)	0.003
Ethnicity, *n* (%)						<0.001
Caucasian	1,350 (75.0%)	324 (72.0%)	358 (79.6%)	357 (79.3%)	311 (69.1%)	
African American	200 (11.1%)	43 (9.6%)	41 (9.1%)	43 (9.6%)	73 (16.2%)	
Hispanic	64 (3.6%)	17 (3.8%)	16 (3.6%)	15 (3.3%)	16 (3.6%)	
Asian	42 (2.3%)	11 (2.4%)	9 (2.0%)	10 (2.2%)	12 (2.7%)	
Native American	38 (2.1%)	9 (2.0%)	7 (1.6%)	5 (1.1%)	17 (3.8%)	
Other/Unknown	106 (5.9%)	46 (10.2%)	19 (4.2%)	20 (4.4%)	21 (4.7%)	
Creatinine-to-albumin, media*n* (IQR)	0.74 (0.43–1.35)	0.29 (0.23–0.36)	0.56 (0.49–0.65)	0.97 (0.84–1.12)	1.95 (1.60–2.72)	<0.001
Albumin, g/dL, median (IQR)	2.5 (2.1–2.9)	2.75 (2.30–3.10)	2.50 (2.20–2.90)	2.40 (2.10–2.80)	2.20 (1.80–2.70)	<0.001
Bilirubin, mg/dL, median (IQR)	0.7 (0.4–1.1)	0.60 (0.40–0.90)	0.70 (0.40–1.00)	0.70 (0.50–1.30)	0.70 (0.50–1.30)	<0.001
BUN, mg/dL, median (IQR)	36 (23–56)	18.00 (12.00–25.00)	29.50 (23.00–41.00)	44.00 (33.25–60.38)	61.00 (45.00–82.00)	<0.001
Creatinine, mg/dL, median (IQR)	1.8 (1.09–3.2)	0.77 (0.62–0.91)	1.42 (1.20–1.70)	2.34 (1.97–2.80)	4.54 (3.66–5.90)	<0.001
Glucose, mg/dL, median (IQR)	208 (142–280)	217.00 (161.00–284.75)	199.50 (117.25–278.75)	216.50 (149.25–279.00)	201.00 (102.25–279.75)	0.006
Heart Rate, beats/min, median (IQR)	112 (96–128.25)	115.00 (101.00–130.00)	111.00 (96.00–128.75)	112.00 (96.00–128.00)	109.00 (92.25–127.00)	0.012
Hematocrit, median (IQR)	30.5 (26.5–34.8)	31.50 (28.20–36.18)	31.00 (27.03–35.30)	30.00 (26.20–33.98)	29.20 (25.20–32.77)	<0.001
MBP, mmHg, median (IQR)	55 (47–118.25)	61.00 (52.00–122.00)	56.00 (48.00–113.75)	52.00 (45.00–68.00)	53.00 (44.00–122.75)	<0.001
Respiratory rate, breaths/min, median (IQR)	31 (14–39)	33.00 (24.00–40.00)	31.00 (16.00–38.00)	32.50 (18.00–39.00)	29.00 (12.00–38.00)	0.004
Sodium, mEq/L, median (IQR)	137 (134–140)	138.00 (135.00–141.00)	138.00 (135.00–141.00)	137.00 (133.00–140.00)	136.00 (132.25–139.00)	<0.001
Temperature, °C, median (IQR)	36.5 (36.2–36.8)	36.60 (36.20–36.90)	36.50 (36.20–36.87)	36.50 (36.10–36.80)	36.40 (36.00–36.70)	<0.001
WBC, 1000/uL, median (IQR)	13.7 (8.8–20)	11.75 (7.93–17.00)	13.70 (9.01–19.52)	14.47 (8.85–21.30)	15.10 (9.67–22.58)	<0.001
Source of infection, *n* (%)						<0.001
Pulmonary	606 (33.7%)	174 (38.7%)	160 (35.6%)	153 (34.0%)	119 (26.4%)	
Urinary	468 (26.0%)	121 (26.9%)	117 (26.0%)	121 (26.9%)	109 (24.2%)	
Gastrointestinal	225 (12.5%)	37 (8.2%)	59 (13.1%)	61 (13.6%)	68 (15.1%)	
Cutaneous/soft tissue	180 (10.0%)	39 (8.7%)	45 (10.0%)	46 (10.2%)	50 (11.1%)	
Gynecologic	5 (0.3%)	2 (0.4%)	0 (0.0%)	2 (0.4%)	1 (0.2%)	
Other/unknown	316 (17.6%)	77 (17.1%)	69 (15.3%)	67 (14.9%)	103 (22.9%)	
Norepinephrine, *n* (%)	430 (23.9%)	72 (16.0%)	103 (22.9%)	120 (26.7%)	135 (30.0%)	<0.001
Insulin, *n* (%)	1,055 (58.6%)	269 (59.8%)	257 (57.1%)	258 (57.3%)	271 (60.2%)	0.693
Statins, *n* (%)	306 (17.0%)	83 (18.4%)	92 (20.4%)	70 (15.6%)	61 (13.6%)	0.0307
Aggressive treatment, *n* (%)	1,346 (74.8%)	302 (67.1%)	312 (69.3%)	359 (79.8%)	373 (82.9%)	<0.001
Septic shock, *n* (%)	402 (22.3%)	69 (15.3%)	79 (17.6%)	122 (27.1%)	132 (29.3%)	<0.001
Tumor, *n* (%)	88 (4.9%)	17 (3.8%)	26 (5.8%)	26 (5.8%)	19 (4.2%)	0.368
Metastatic cancer, *n* (%)	60 (3.3%)	17 (3.8%)	17 (3.8%)	19 (4.2%)	7 (1.6%)	0.108
Lymphoma, *n* (%)	12 (0.7%)	2 (0.4%)	2 (0.4%)	6 (1.3%)	2 (0.4%)	0.285
Leukemia, *n* (%)	36 (2.0%)	9 (2.0%)	7 (1.6%)	11 (2.4%)	9 (2.0%)	0.824
CKD, *n* (%)	220 (12.2%)	10 (2.2%)	39 (8.7%)	62 (13.8%)	109 (24.2%)	<0.001
Cirrhosis, *n* (%)	72 (4.0%)	15 (3.3%)	18 (4.0%)	19 (4.2%)	20 (4.4%)	0.847
Hepatic failure, *n* (%)	47 (2.6%)	6 (1.3%)	16 (3.6%)	11 (2.4%)	14 (3.1%)	0.175
COPD, *n* (%)	85 (4.7%)	31 (6.9%)	19 (4.2%)	23 (5.1%)	12 (2.7%)	0.0253
APE, *n* (%)	5 (0.3%)	0 (0.0%)	2 (0.4%)	2 (0.4%)	1 (0.2%)	0.763
Myocardial infarction, *n* (%)	12 (0.7%)	3 (0.7%)	1 (0.2%)	4 (0.9%)	4 (0.9%)	0.583
CHD, *n* (%)	70 (3.9%)	14 (3.1%)	15 (3.3%)	19 (4.2%)	22 (4.9%)	0.487
Ischemic stroke, *n* (%)	8 (0.4%)	3 (0.7%)	3 (0.7%)	1 (0.2%)	1 (0.2%)	0.654
Hyperlipidemia, *n* (%)	21 (1.2%)	7 (1.6%)	5 (1.1%)	8 (1.8%)	1 (0.2%)	0.136
Hypertension, *n* (%)	93 (5.2%)	31 (6.9%)	24 (5.3%)	22 (4.9%)	16 (3.6%)	0.157
Coagulation defects, *n* (%)	44 (2.4%)	6 (1.3%)	13 (2.9%)	11 (2.4%)	14 (3.1%)	0.315
Thyroid disease, *n* (%)	34 (1.9%)	8 (1.8%)	13 (2.9%)	10 (2.2%)	3 (0.7%)	0.0955
Immune suppression, *n* (%)	101 (5.6%)	25 (5.6%)	33 (7.3%)	27 (6.0%)	16 (3.6%)	0.1
Intubated, *n* (%)	367 (20.4%)	80 (17.8%)	88 (19.6%)	97 (21.6%)	102 (22.7%)	0.273
Vent, *n* (%)	553 (30.7%)	129 (28.7%)	133 (29.6%)	156 (34.7%)	135 (30.0%)	0.205
Dialysis, *n* (%)	149 (8.3%)	0 (0.0%)	1 (0.2%)	22 (4.9%)	126 (28.0%)	<0.001
LOS ICU, days, median (IQR)	2.78 (1.78–5.17)	2.45 (1.63–4.21)	2.59 (1.77–4.21)	3.01 (1.85–5.59)	3.40 (1.91–6.10)	<0.001
ICU death, *n* (%)	183 (10.2%)	15 (3.3)	32 (7.1)	59 (13.1)	77 (17.1)	<0.001

IQR, interquartile range; BMI, body mass index; WBC, white blood cell; BUN, blood urea nitrogen; MBP, mean blood pressure; COPD, chronic obstructive pulmonary disease; CHD, coronary heart disease; CKD, chronic kidney disease; APE, acute pulmonary embolism; LOS, length of stay.

### 3.2 Elevated CAR levels are associated with increased 28-day mortality in the ICU

**Figure 2:**
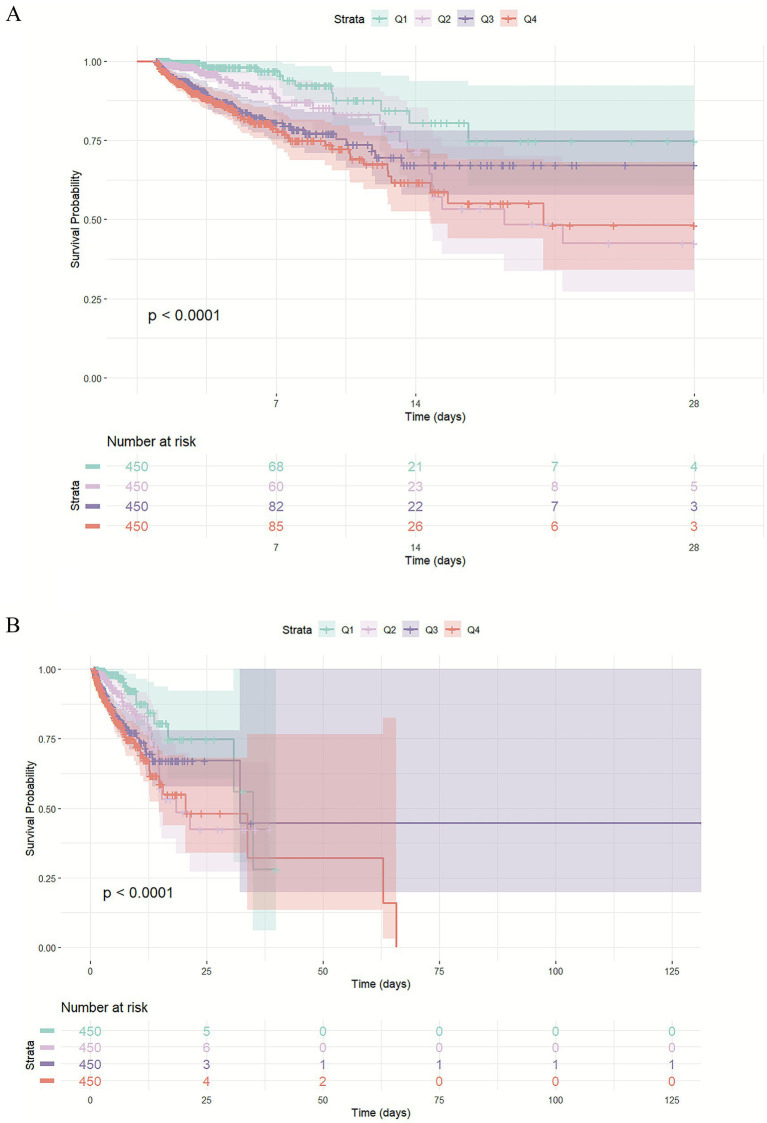
The Kaplan–Meier curves depict the cumulative probability of 28-day ICU mortality **(A)** and overall mortality **(B)** among different groups.

**Figure 3:**
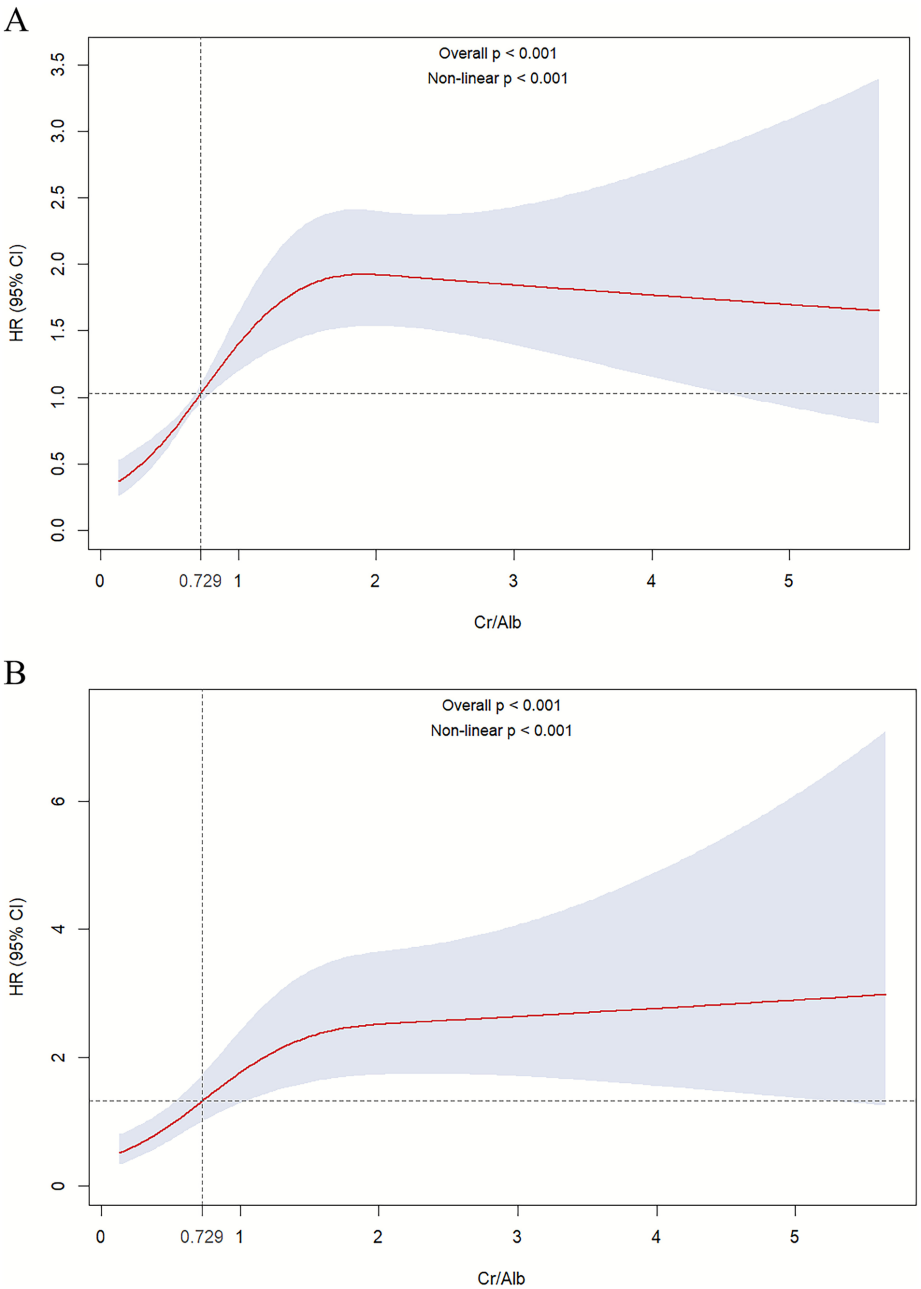
Restricted cubic spline regression analysis of serum creatinine-to-albumin ratio and 28-day all-cause ICU mortality. Panels **(A,B)** illustrate the nonlinear association between serum creatinine-to-albumin ratio (Cr/Alb) and the risk of 28-day ICU mortality in univariate and multivariate adjusted models, respectively. The solid red line represents the estimated hazard ratio (HR), and the shaded area indicates the 95% confidence interval. The dashed vertical line indicates the threshold above which the serum creatinine-to-albumin ratio is associated with a rapid increase in mortality risk.

Kaplan–Meier survival curve analysis (Figure 2) revealed that patients with higher CAR quartiles had significantly higher risks of 28-day ICU mortality as well as all-cause mortality. To further quantify this relationship, Cox proportional hazards regression analyses were conducted (Table 2). When CAR was treated as a continuous variable, each unit increase in CAR was independently associated with elevated 28-day ICU mortality in both the unadjusted Model 1 [HR 1.27 (95% CI 1.15–1.41), *p* < 0.001] and the fully adjusted Model 2 [HR 1.36 (95% CI 1.19–1.55), *p* < 0.001]. When analyzed by quartiles, compared with the lowest quartile (Q1), patients in the higher CAR quartiles exhibited markedly increased mortality risk across all models, with the highest quartile (Q4) demonstrating [unadjusted Model 1: HR 4.55 (95% CI 2.52–8.21), *p* < 0.001; adjusted Model 2: HR 4.68 (95% CI 2.55–8.61), *p* < 0.001], and a significant dose–response trend of increasing risk across quartiles (*P* for trend < 0.001). Furthermore, restricted cubic spline analysis confirmed a nonlinear association between CAR and 28-day ICU mortality (*p* < 0.001) (Figure 3), indicating that the risk increases nonlinearly with rising levels. The RCS regression identified a potential risk threshold, whereby higher CAR levels above 0.729 were associated with significantly increased risk of ICU mortality.

**Table 2:** Cox regression model of serum CAR and 28-day all-cause mortality in the ICU.

CAR		Model 1			Model 2	
	HR (95% CI)	*P*-value	*P* for trend	HR (95% CI)	*P*-value	*P* for trend
Continuous variable per 1 unit	1.27 (1.15–1.41)	<0.001		1.36 (1.19–1.55)	<0.001	
Quartile			<0.001			<0.001
Q1 (N = 450)	Reference			Reference		
Q2 (N = 450)	2.41 (1.26–4.60)	0.007		2.47 (1.29–4.76)	0.006	
Q3 (N = 450)	3.76 (2.06–6.87)	<0.001		3.57 (1.93–6.62)	<0.001	
Q4 (N = 450)	4.55 (2.52–8.21)	<0.001		4.68 (2.55–8.61)	<0.001	

Model 1: unadjusted.

Model 2: adjusted for age, ethnicity, source of infection, norepinephrine, hypertension, acute pulmonary embolism, white blood cell, temperature, respiratory rate, bilirubin, metastatic cancer, leukemia.

**Figure 4:**
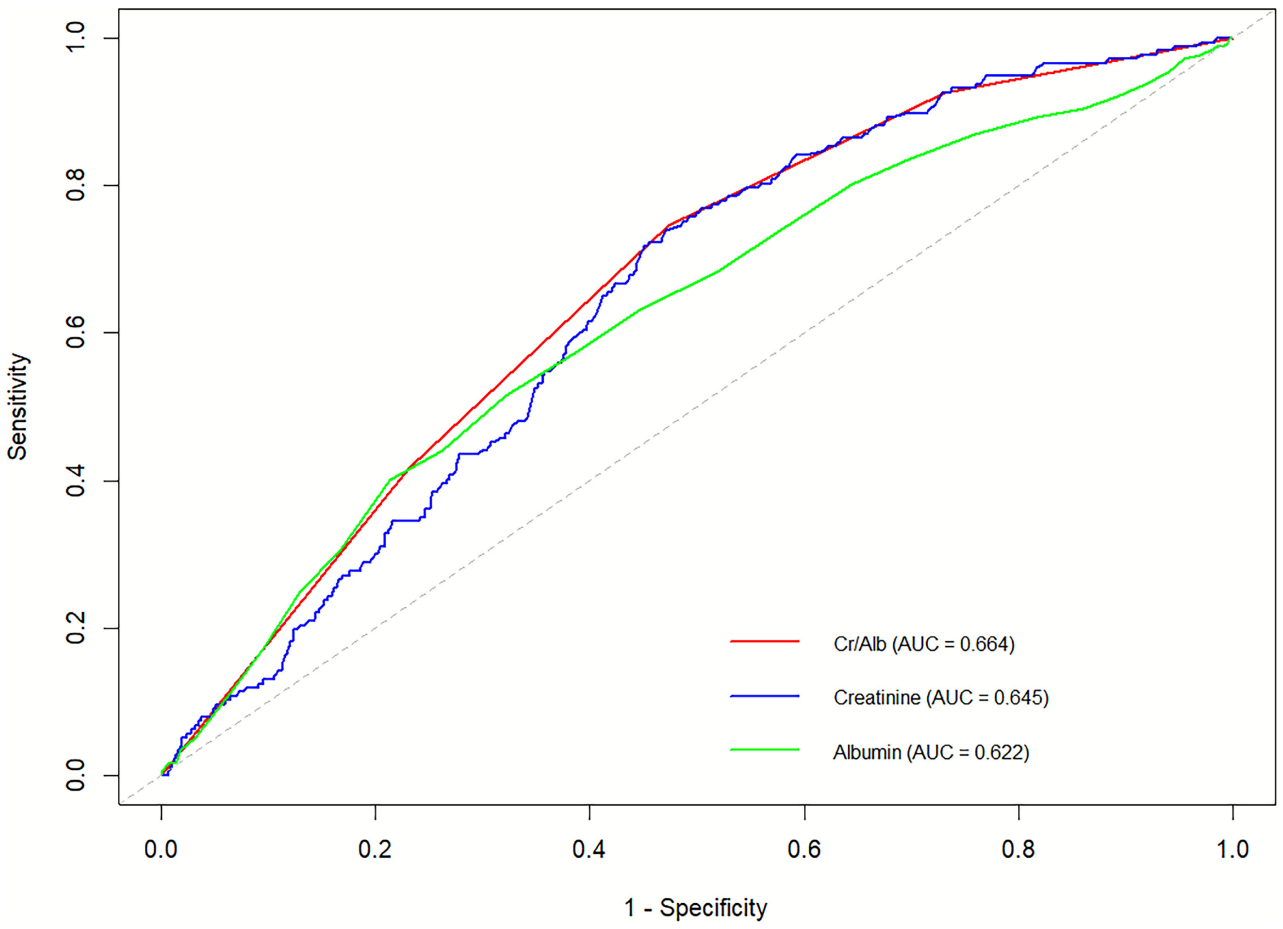
Receiver operating characteristic (ROC) curve of serum creatinine-to-albumin ratio (Cr/Alb) for predicting 28-day ICU mortality.

To assess whether CAR outperforms serum creatinine and albumin alone, we generated a receiver operating characteristic (ROC) curve (Figure 4). The analysis demonstrated that CAR had superior predictive performance for 28-day mortality risk following ICU admission, with an area under the curve (AUC) of 0.664, surpassing that of the individual markers.

### 3.3 Subgroup analysis of clinical outcomes in patients with sepsis and diabetes using CAR

**Figure 5:**
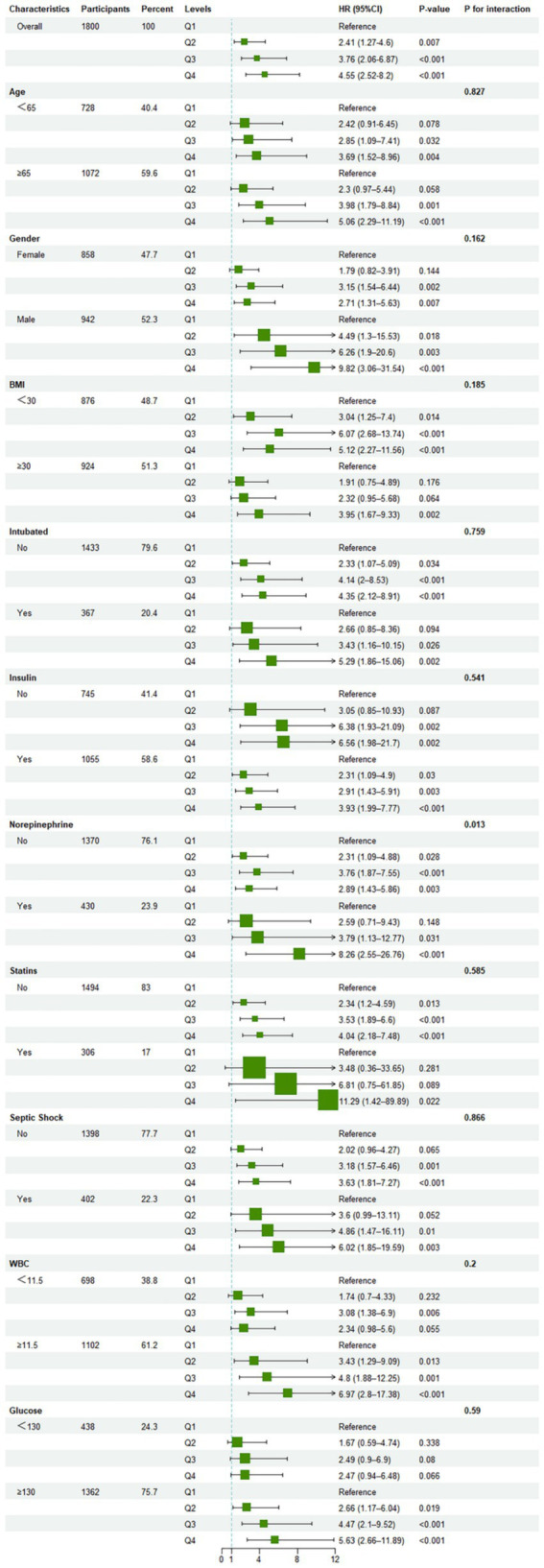
Forest plot of hazard ratios for 28-day ICU mortality across different subgroups.

A stratified analysis of the relationship between CAR and 28-day ICU mortality (Figure 5) showed a positive correlation between CAR and increased mortality risk across all subgroups. Notably, the predictive power of CAR was particularly pronounced in patients receiving norepinephrine therapy [norepinephrine-free group HR 2.89 (95% CI 1.43–5.86) vs. norepinephrine-treated group HR 8.26 (95% CI 2.55–26.76), P for interaction = 0.013].

### 3.4 Relationship between CAR and ICU length of stay

Multivariate linear regression analysis indicated that higher CAR was associated with prolonged ICU length of stay (*β* = 1.06, *p* = 0.006) (Table 3).

**Table 3:** Relationship between serum CAR and ICU length of stay.

CAR	Model 1				Model 2			
	Estimate	S.E	*t*-value	*P*-value	Estimate	S.E	*t*-value	*P*-value
Continuous variable per 1 unit^a^	0.3	0.14	2.12	0.034	0.22	0.14	1.54	0.12
Quartile^a^								
Q1	Reference				Reference			
Q2	0.04	0.37	0.1	0.923	0.06	0.37	0.16	0.872
Q3	1.14	0.38	2.99	0.003	1.06	0.38	2.77	0.006
Q4	0.92	0.39	2.39	0.017	0.78	0.39	2.00	0.046

Model 1: unadjusted.

Model 2: adjusted for age, ethnicity, source of infection, norepinephrine, hypertension, acute pulmonary embolism, white blood cell, temperature, respiratory rate, bilirubin, metastatic cancer, leukemia.

^a^The association between serum creatinine-to-albumin ratio and ICU length of stay was analyzed in the cohort of ICU survivors (*N* = 1,617).

## 4 Discussion

The findings of this study demonstrate a significant positive association between the CAR and 28-day all-cause mortality in ICU patients with sepsis and diabetes. Kaplan–Meier survival curve analysis showed that elevated CAR levels were associated with increased 28-day mortality risk. Multivariate Cox regression further established CAR as an independent predictor of 28-day mortality. Restricted cubic spline analysis revealed that this association may be a complex nonlinear relationship. Subgroup analysis highlighted a particularly strong association in patients receiving norepinephrine therapy, where elevated CAR was linked to a markedly higher mortality risk compared with those not requiring norepinephrine support. Furthermore, higher CAR levels were associated with an extended ICU length of stay. Notably, the predictive performance of CAR for 28-day ICU mortality, as assessed by the AUC, outperformed that of serum creatinine or albumin alone.

The results of the present study align with prior research demonstrating the prognostic significance of the CAR in critically ill populations. For instance, a study investigating the predictive value of CAR and the lactate to albumin ratio (LAR) for sepsis-associated persistent severe acute kidney injury reported that (28), compared with patients without diabetes, those with diabetes exhibited elevated CAR levels and a substantially higher risk of acute kidney injury, with a significant interaction effect [7.42 (95% CI 4.22–13.03), *P* for interaction = 0.021]. Similarly, CAR’s predictive utility for 28-day mortality has been confirmed in critically ill patients following cardiac surgery (29), where patients with diabetes demonstrated a markedly increased mortality risk [2.14 (95% CI 1.21–3.77), *p* = 0.009]. These findings imply that the prognostic value of CAR may be disease-specific (23), exhibiting enhanced predictive capacity in patients with sepsis and diabetes. Moreover, the association between CAR and 28-day all-cause mortality in ICU patients with sepsis and diabetes appears to be nonlinear and complex, as revealed by restricted cubic spline analysis. This nuanced relationship facilitates the identification of critical CAR thresholds, which may help stratify high-risk patients who could benefit from early preventive interventions. ROC curve analyses corroborate existing literature, suggesting that CAR possesses superior sensitivity and specificity relative to individual biomarkers, thereby providing a more accurate prediction of 28-day mortality. However, an AUC of 0.664 indicates moderate predictive performance, which aligns well with evaluations of CAR in similar studies. Its precise clinical prognostic value still requires confirmation through additional prospective research. Currently, CAR is best positioned as a valuable complementary or adjunctive biomarker, ideally used in combination with established scoring systems such as SOFA, to improve risk stratification and provide a foundation for developing future multidimensional prognostic models (22, 24, 28). Specifically, serial monitoring of CAR during the ICU stay may be considered, thereby complementing the organ dysfunction assessment provided by SOFA scores and allowing for the evaluation of treatment response based on CAR trajectories. In the future, integrating CAR with patients’ clinical information and established scoring systems such as SOFA could enable the construction of combined predictive models, with predefined risk alert thresholds to facilitate enhanced resource allocation and early intervention in subgroups identified as being at elevated risk of mortality. Subgroup analyses demonstrated that the prognostic value of CAR remains consistent across stratifications by gender, age, BMI, WBC, and blood glucose levels, and was unaffected by concomitant use of statins or insulin. Interestingly, CAR exhibited a significantly stronger predictive capacity for mortality in patients receiving norepinephrine therapy, with a statistically significant interaction effect. This suggests that CAR may hold particular clinical utility for risk stratification among critically ill patients. Furthermore, higher CAR values were associated with prolonged ICU stay, reinforcing its potential as a prognostic biomarker in the sepsis-diabetes population. These associations appear to be independent of traditional demographic variables and may reflect underlying pathophysiological mechanisms central to disease progression.

Although the exact pathophysiological mechanisms linking elevated CAR to mortality risk in patients with sepsis and diabetes remain incompletely understood and largely speculative, the observed strong association between CAR and mortality risk in patients with sepsis and diabetes may reflect the combined impact of kidney injury and nutritional-inflammatory imbalance. First, the pathogenesis of sepsis is closely related to the pro-inflammatory-anti-inflammatory imbalance following host infection (30), while the chronic inflammatory milieu and immune dysregulation characteristic of diabetes could amplify the inflammatory cascade inherent to sepsis (3). Moreover, sepsis-induced stress hyperglycemia might exacerbate underlying diabetes and intensify oxidative stress-mediated tissue damage (31). Second, the accumulation of inflammatory mediators during sepsis can directly injure endothelial cells (32). Concurrently, diabetes-associated hyperglycemia and oxidative stress are thought to further impair endothelial function by reducing nitric oxide synthesis and upregulating adhesion molecule expression, thereby potentially perpetuating endothelial dysfunction (33). Additionally, endothelial microthrombosis and endocrine disturbances related to diabetes may contribute to metabolic derangements (34). Third, it is hypothesized that the intensified inflammatory response and endothelial injury in patients with sepsis and diabetes could precipitate microcirculatory dysfunction, impaired renal perfusion, and aggravated sepsis-associated acute kidney injury, potentially resulting in elevated serum creatinine levels (35). Persistent elevation of inflammatory mediators, due to inadequate clearance, might sustain systemic inflammation. Under severe inflammatory and stress conditions, hepatic albumin synthesis often decreases, while albumin catabolism increases. Concurrently, renal insufficiency can promote albumin loss through increased leakage, commonly leading to reduced serum albumin concentrations (36, 37). Collectively, these factors are proposed to contribute to an elevated CAR, which thereby integrates signals related to sepsis severity in patients with diabetes and shows an association with increased mortality risk. Fourth, norepinephrine administration typically indicates septic shock, characterized by maximal systemic inflammation, endothelial damage, microcirculatory failure, and organ dysfunction (38). Moreover, norepinephrine’s potent *α*-adrenergic agonist effects may exacerbate vasoconstriction, possibly further compromising renal ischemia and hypoxia (39). We speculate that at this stage, the pathophysiological processes reflected by CAR may peak, which might explain why markedly elevated CAR levels predict mortality. In summary, the pathophysiology of sepsis complicated by diabetes is complex. These interpretations are plausible based on current literature but remain speculative, as our observational study cannot establish causality. Further mechanistic research, including prospective studies, *in vitro* experiments, animal models, and detailed pathway analyses, is warranted to elucidate these dynamic interactions, validate the hypothesized mechanisms, and identify novel therapeutic targets for high-risk patients.

The study has several limitations. First, its retrospective design precludes the establishment of causal relationships. Although multivariate regression and subgroup analyses were employed to adjust for confounding variables, key factors could not be fully accounted for, and residual confounding bias may persist. It should also be noted that this study employed single imputation using the median and mode to handle missing data. Although this method is straightforward and preserves the overall sample size, it may underestimate data variability and potentially introduce bias by attenuating associations between variables. Despite only seven variables having missing data, each with a missing proportion of less than 10%, future prospective designs are still needed to reduce missingness and mitigate these risks. Second, only baseline CAR levels were assessed, without monitoring their dynamic changes throughout intensive care treatment. Although this study did not distinguish between type 1 and type 2 diabetes, both types are characterized by significant renal impairment and inflammatory contributions. Moreover, the prognostic association of the CAR remained largely consistent across relevant subgroups in this cohort; therefore, our findings retain substantial clinical relevance. Future investigations could further explore the influence of diabetes subclassification on the predictive performance of the CAR. Third, the analysis was restricted to a single database, primarily sourced from medical institutions in the United States, with patients predominantly Caucasian and clinical practices potentially influenced by the U.S. healthcare system. Therefore, direct generalization of the study findings to other ethnic groups (e.g., Asian or African populations) or different healthcare systems (e.g., in Europe or developing countries) should be interpreted with caution. Future studies should prioritize multicenter, international external validation to evaluate the predictive performance of the CAR across diverse populations and healthcare settings, and to further confirm its clinical applicability. In addition, prospective studies and corresponding mechanistic investigations are needed to establish causal relationships and to provide more reliable evidence-based guidance for clinical practice.

## 5 Conclusion

This study demonstrated that the CAR was significantly associated with 28-day all-cause mortality in ICU patients with sepsis and diabetes. As a readily obtainable biomarker, the CAR may serve as a valuable tool for early assessment of disease severity and prognosis in this high-risk population. Nevertheless, further research is warranted to validate its clinical utility and to explore its potential role in guiding therapeutic decision-making.

## Data Availability

Publicly available datasets were analyzed in this study. This data can be found at: https://eicu-crd.mit.edu/.
